# Gene expression profiling analysis to investigate the role of remote ischemic postconditioning in ischemia-reperfusion injury in rats

**DOI:** 10.1186/s12864-019-5743-9

**Published:** 2019-05-09

**Authors:** Zanxin Wang, Junmin Wen, Chuzhi Zhou, Zhiwei Wang, Minxin Wei

**Affiliations:** 1 Department of Cardiac Surgery, Fuwai Hospital Chinese Academy of Medical Sciences Shenzhen, 12 Langshan Road, Nanshan District, Shenzhen, 518057 Guangdong Province People’s Republic of China; 2 Department of Cardiac Surgery, Shenzhen Sun Yat-sen Cardiovascular Hospital, Shenzhen, People’s Republic of China; 3 Department of Cardiovascular Surgery, Tianjin Medical University General Hospital, Tianjin, People’s Republic of China; 4 Department of Intensive Care, Fuwai Hospital Chinese Academy of Medical Sciences Shenzhen, Shenzhen, Guangdong People’s Republic of China; 5 Department of Intensive Care, Shenzhen Sun Yat-sen Cardiovascular Hospital, Shenzhen, People’s Republic of China

**Keywords:** IRI, RPostC, Apoptosis, Gene, ncRNAs

## Abstract

**Background:**

Blood flow restoration is a definitive therapy for salvaging the myocardium following ischemic injury. Nevertheless, the sudden restoration of blood flow to the ischemic myocardium can induce ischemia-reperfusion injury (IRI).

**Results:**

Herein, we investigated the cardioprotective effect of remote ischemic postconditioning (RPostC) through our in vivo rat model of myocardial IRI. The study included three groups: the control group, the IRI group, and the IRI + RPostC group. Ischemia-reperfusion treatment led to an increase in the myocardial infarction area, which was inhibited by RPostC. In contrast to that in the control group, the myocardial apoptosis level was enhanced in the IRI group, whereas RPostC treatment decreased IRI-induced cellular apoptosis. Affymetrix Rat Gene 2.0 ST chip data identified a total of 265 upregulated genes and 267 downregulated genes between the IRI and IRI + RPostC groups. A group of differentially expressed noncoding RNAs (ncRNAs), such as MTA_TC0600002772.mm, MTA_TC1300002394.mm, U7 small nuclear RNA (Rnu7) and RGD7543256_1, were identified. Gene Ontology (GO) enrichment analysis indicated that the positive regulation of some molecular functions, such as GTPase activity, GTP binding, cyclic-nucleotide phosphodiesterase activity and cytokine activity, may contribute to the cardioprotective role of RPostC. Moreover, pathway enrichment analysis using the Kyoto Encyclopedia of Genes and Genomes (KEGG) suggested the potential implication of the TNF signaling pathway and Toll-like receptor signaling pathway. Global signal transduction network analysis, co-expression network analysis and quantitative real-time polymerase chain reaction analysis further identified several core genes, including *Pdgfra*, *Stat1, Lifr* and *Stfa3.*

**Conclusion:**

Remote ischemic postconditioning treatment can decrease IRI-mediated myocardial apoptosis by regulating multiple processes and pathways, such as GTPase activity, cytokine activity, and the TNF and Toll-like receptor signaling pathways. The potential role of the above ncRNAs and core genes in IRI-induced cardiac damage merits further study as well.

**Electronic supplementary material:**

The online version of this article (10.1186/s12864-019-5743-9) contains supplementary material, which is available to authorized users.

## Background

Ischemia-reperfusion injury (IRI) is a phenomenon in which cell metabolism and structural damage are aggravated when blood flow is restored after myocardial ischemia and reperfusion [1–3]. Reperfusion treatment can open the infarct-related blood vessels quickly and fully restore the blood perfusion of the ischemic myocardium, rescuing the dying myocardium. Nevertheless, during the recovery of coronary blood flow, myocardial IRI can induce myocardial contraction disorders, myocardial infarct size expansion and other adverse consequences [2–4]. Our prior clinical studies reported that IRI is accompanied by different degrees of inflammatory factors, which exhibit a great influence on prognosis [5]. Hence, it is important to further investigate the mechanism underlying myocardial IRI from different perspectives and how to effectively minimize this damage through human intervention to protect the heart tissue.

Thus far, accumulating data support the association between myocardial IRI and cell stress and apoptosis; nevertheless, ischemic preconditioning, ischemic postconditioning (PostC), remote ischemic postconditioning (RPostC) and other protective measures beyond timely reperfusion are reported to reduce the myocardial damage caused by ischemia reperfusion [6–12]. As a short-term reperfusion/ischemia treatment, PostC is implemented prior to continuous reperfusion after myocardial ischemia [6–8, 10]. RPostC refers to the protective effect seen upon the transient ischemic treatment of distant organs or tissues before the restoration of blood flow to the ischemic area [9, 13–16]. Because RPostC avoids the clamping of coronary vessels, it achieves this myocardial protective effect through a simple and noninvasive intervention for the ischemic postconditioning of distant position and thus represents an optimistic prospect for clinical application [9, 13–16]. Nevertheless, there is insufficient experimental evidence for the mechanisms involved in the protection of the myocardium by RPostC.

In the present study, we aimed to investigate the transcriptional changes involved in cardioprotection by RPostC in rats. Based on an in vivo rat model of IRI and RPostC, a series of assays, including gene expression profiling, functional pathway analysis, global signal transduction network analysis, co-expression network analysis and quantitative polymerase chain reaction (PCR) analysis, were conducted.

## Methods

### IRI and IRI + RPostC model of rats

Referring to the relative literature [17, 18], we established our rat model of IRI and RPostC treatment. A total of 24 specific pathogen-free grade Wistar male rats (Tianjin Shanchuan Red Experimental Animal Technology Co., Ltd.) were used; the rats were housed under standard conditions of temperature (22 °C ± 2 °C) and humidity (40% ~ 70%) and a 12 h/12 h light/dark cycle. Rats were randomly assigned to one of three groups: the control group, the IRI group or the IRI + RPostC group. The rats were anesthetized by 1% sodium pentobarbital. After endotracheal intubation, a small animal ventilator was connected. We cut the skin longitudinally along the sternum, bluntly separated the muscles with hemostatic forceps, exposed the third to fifth ribs on the left side of the sternum, and opened the chest in the fourth intercostal space on the left side of the chest. Then, we cut the pericardium and fixed the thorax with a homemade hook to fully expose the heart. The needle was inserted into the left atrial appendage and placed under the conus of the pulmonary artery. The anterior descending branch was ligated with a 6/0 medical suture for 30 min and then loosened, and the thoracic cavity was sutured layer by layer.

In the control group, we did not block the blood vessels of the anterior descending branch after threading, and the chest was closed after 60 min of threading. In the IRI group, the left anterior descending coronary artery (LAD) was blocked and opened 45 min later. After 15 min of stabilization, the chest was closed. In the IRI + RPostC group, after 45 min of LAD occlusion, we performed the RPostC treatment. The left femoral artery (LFA) was clamped for 30 s and then opened for 30 s. This process was repeated three times, and the chest was closed after 15 min of stabilization. We performed these experiments under the guidelines of animal experiments at Tianjin Medical University (2014 revision). Ethical approval for all experimental procedures was granted by the Animal Ethical and Welfare Committee at Tianjin Medical University.

### 2,3,5-triphenylte-trazolium chloride (TTC) staining assay

After myocardial ischemia for 45 min and reperfusion for 24 h, the chest of the rat was opened. After quick freezing for 15 min, we obtained five pieces of myocardial tissue with a thickness of approximately 1 mm along the long axis of the vertical heart. Next, we immediately clamped them between two slides to gently flatten them and prevent shrinkage. The samples were then placed in a 1% TTC-phosphate buffer saline (PBS) solution at 37 °C in a constant temperature water bath for 15 min and then in 4% formaldehyde solution for 12 h. The size of the myocardial infarction area, which was white or pale, was measured by ImageJ 2X software.

### Terminal deoxynucleotidyl transferase (TdT)-mediated dUTP nick end labeling (TUNEL) assay

We used a TUNEL kit (Roche, Germany) according to the manufacturer’s instructions to analyze the cardiomyocyte apoptosis level after reperfusion. The heart tissues were washed with PBS solution (pH 7.4). Then, the atrium was removed, and the ventricular part below the coronary artery ligation point was preserved. The vertical section of the ventricle was transversely cut into 4 ~ 6 pieces of 1 ~ 2-mm thick myocardial slices. The percentage of TUNEL-positive cells was determined by ImageJ 2X software.

### RNA microarray detection

We utilized TRIzol reagent (Life Technologies) and the RNeasy Mini kit (Qiagen) to extract and purify total RNA from the cardiac left ventricle (CLV) tissue of the above three groups. Then, we used the Ambion WT Expression Kit (Affymetrix) and GeneChip WT Terminal Labeling and Controls Kit (Affymetrix) to obtain cDNA and cRNA products. Next, an Affymetrix Rat Gene 2.0 ST chip was used to hybridize the fragmented cRNA product. After staining with the GeneChip Fluidics Station 450, we applied the Affymetrix® GeneChip Command Console and robust multichip analysis algorithms to scan the microarrays and analyze the data, including Gene Ontology (GO) enrichment analysis and functional pathway analysis using the Kyoto Encyclopedia of Genes and Genomes (KEGG), as reported previously [19, 20]. Based on the GO database, we obtained the gene function annotation of the differential genes detected above and then calculated the significance level (*P* value) and false positive rate (FDR) of each function by Fisher’s exact test and multiple comparison test. Thus, the significant function of the gene was screened out, and a *P* value< 0.05 was considered significant in screening. The series entry (GSE122020, http://www.ncbi.nlm.nih. gov/geo/query/acc.cgi? acc = GSE122020) in the National Center for Biotechnology Information (NCBI) is provided.

### Network analysis

According to the differentially expressed gene data, we also performed a global signal transduction network analysis to show the core genes, which have a strong correlation with other genes and play an essential role in the signaling network. In addition, based on the normalized signal intensity of RNA expression, we performed co-expression network analysis to detect potential correlations among mRNAs and identify the core genes by the degree of differences. We also performed a Venn diagram analysis (http://bioinformatics.psb.ugent.be/webtools/Venn/) to identify the common genes between the above two networks under the different limitation of degree value.

### Quantitative real-time polymerase chain reaction assay (qPCR)

Based on the cDNA synthesized, we performed a StepOne Real-Time PCR System (Applied Biosystems) PCR amplification using FastStart Universal SYBR Green Master Mix (Roche Diagnostics) and the relative primers. The detailed primer sequence information is listed in Additional file 1: Table S1. The standard curve was used to assess the amplification efficiency. The melting curve assay was performed to analyze the amplification specificity. PCR reaction settings: pre-incubation step (95 °C, 15 min); amplification step (95 °C, 10 s; 60 °C, 20 s; 72 °C, 31 s; 40 cycles); melting curve step (95 °C, 15 s; 72 °C, 15 s; 95 °C, 15 s). The 2^-ΔΔCT^ method was applied to calculate the relative transcript levels of candidate genes [21]. The relative expression of Glyceraldehyde-3-Phosphate Dehydrogenase (*Gapdh*) was utilized to normalize the expression of tested genes, including phospholipase C, beta 4 (*Plcb4)*, platelet derived growth factor receptor alpha (*Pdgfra)*, chemokine (C-C motif) receptor 1-like 1 (*Ccr1l1*), signal transducer and activator of transcription 1(*Stat1)*, Jun proto-oncogene, AP-1 transcription factor subunit (*Jun*), Hemoglobin Subunit Alpha 2(*Hba2*), similar to Glutathione S-transferase A1 (GTH1) (HA subunit 1) (GST-epsilon) (GSTA1–1) (GST class-alpha) (*LOC501110*), stefin A3 (*Stfa3),* CASP8 and FADD-like apoptosis regulator (*Cflar*), leukemia inhibitory factor receptor alpha (*Lifr*), and glutathione S-transferase, mu 5 (*Gstm5)*.

### Statistical analysis

We performed one-way analysis of variance (ANOVA) / least significant difference (LSD) test or independent sample Student’s t-test using BM SPSS Statistics 20 Software. When the *P* value was < 0.05, the differences were considered significant.

## Results

### Effect of RPostC treatment on myocardial infarct size in an IRI rat model

**Fig. 1:**
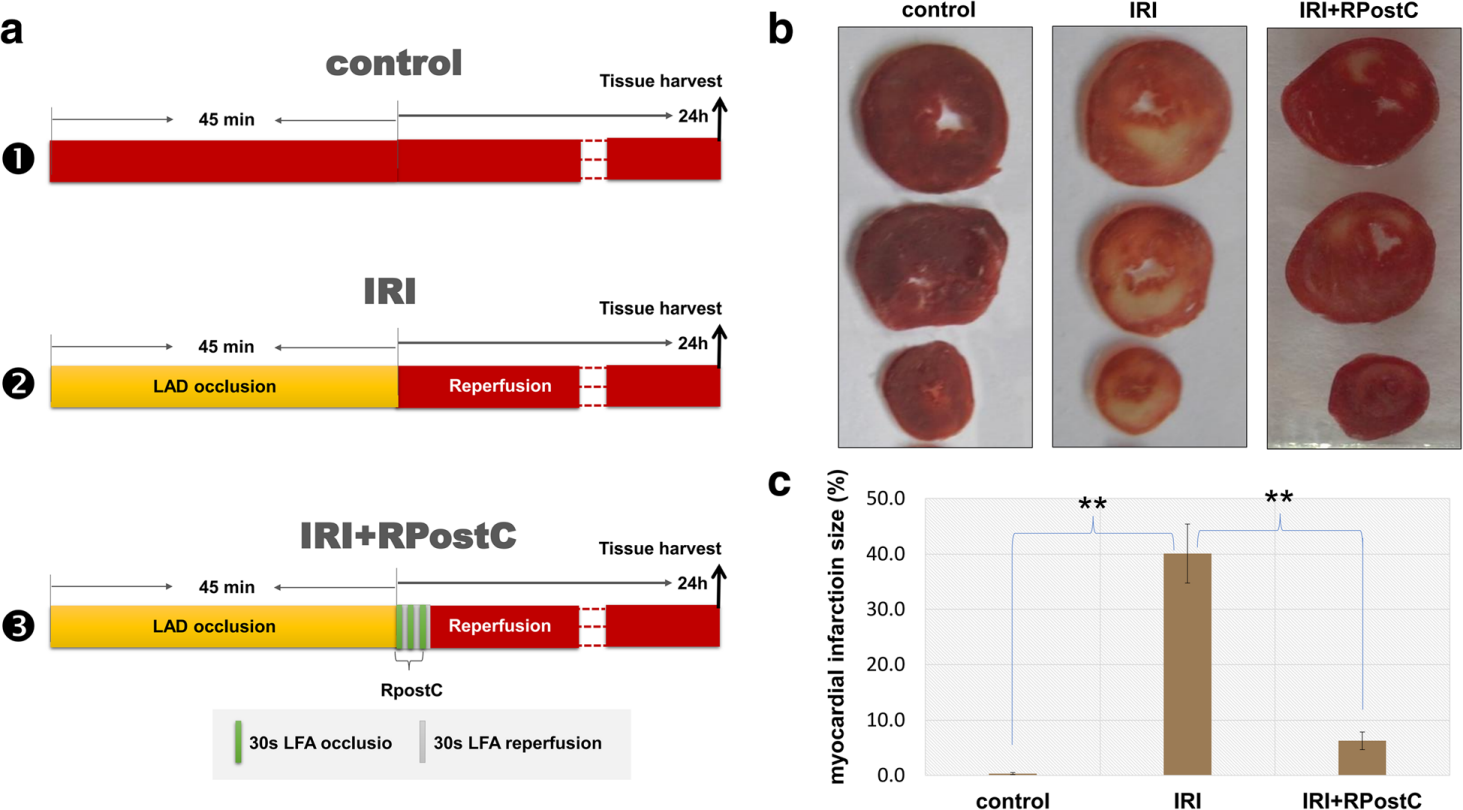
Rat model of IRI and RPostC treatment and myocardial infarction analysis by TTC staining. **a** Schematic diagram of the rat models, including control, IRI and IRI + RpostC; LAD, left anterior descending coronary artery; LFA, left femoral artery. **b** A histological TTC staining assay was then performed to measure the myocardial infarct size. The myocardial infarction images of three groups were shown. **c** ANOVA-LSD test was also performed for the statistical analysis. Significant difference was indicated: **, *P* < 0.01

To gain insight into the potential molecular mechanism of remote ischemic postconditioning (RPostC) in ischemia/reperfusion injury (IRI), we first established an in vivo rat model of myocardial ischemia reperfusion. As shown in Fig. 1a, we did not block the blood vessels in the anterior descending branch of some rats, which were included in the study as controls. We performed RPostC treatment by three cycles of 30 s left femoral artery (LFA) occlusion/30 s reperfusion in rats that underwent IRI, which was induced by 45 min of left anterior descending coronary artery (LAD) occlusion.

Next, we carried out a histological TTC staining assay to preliminarily evaluate the impact of the ischemia/reperfusion process or RPostC treatment in rats suffering from myocardial ischemia-reperfusion injury. TTC staining was utilized to measure the myocardial infarct size. As shown in Fig. 1 b-c, we observed an increased myocardial infarction size in the IRI group compared to the control group (*P* < 0.01). Nevertheless, an increased myocardial infarction area was attenuated when IRI rats were subjected to RPostC treatment in the IRI + RPostC group (Fig. 1 b-c, *P* < 0.01). This finding indicated that RPostC treatment exhibited myocardial protection against ischemia/reperfusion injury in rats.

### Impact of RPostC treatment on the apoptotic level of cardiomyocytes in an IRI rat model

**Fig. 2:**
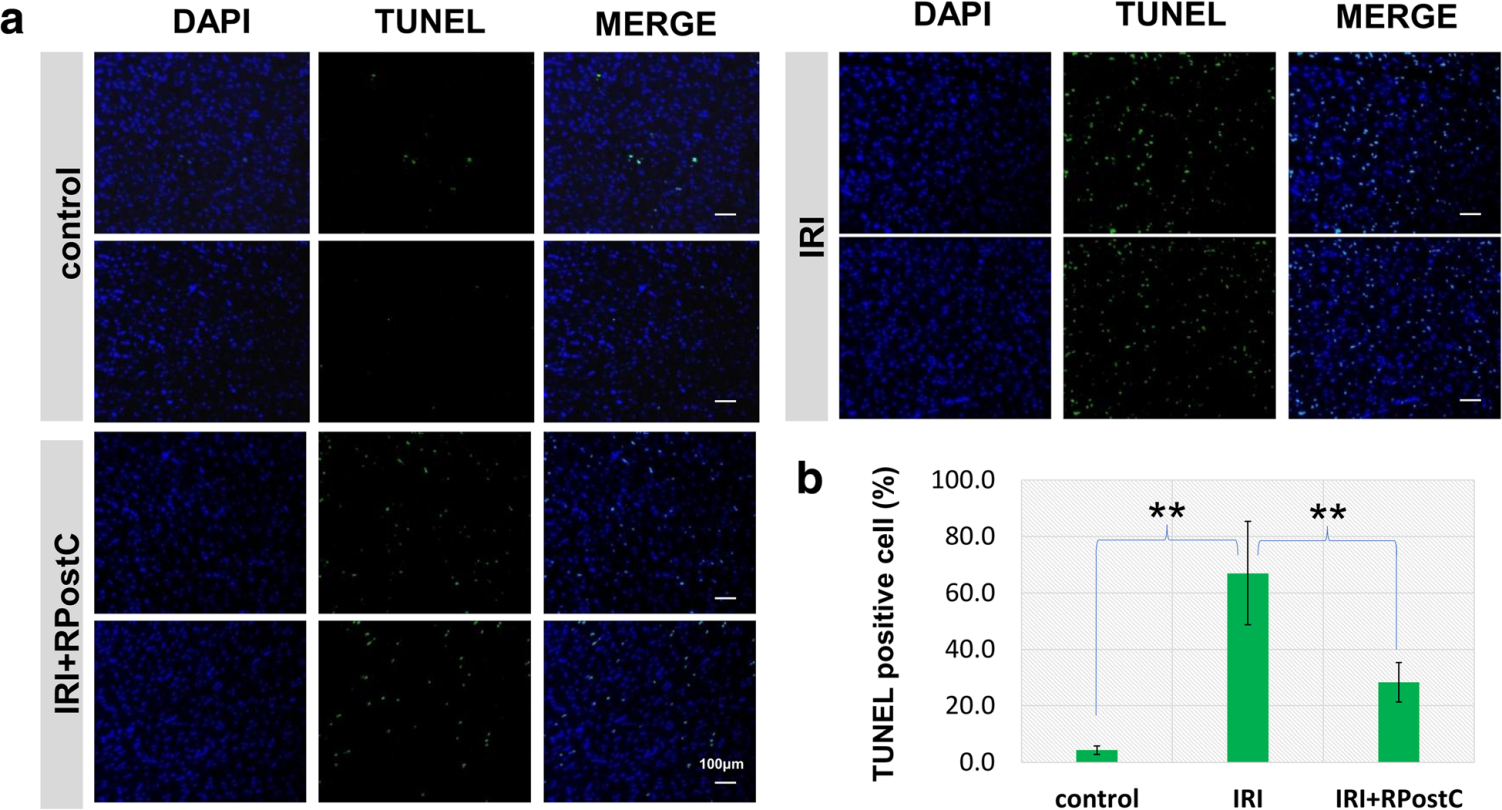
Cardiomyocyte apoptosis analysis by TUNEL assay. **a** A TUNEL assay was performed to analyze the effect of RPostC treatment on the level of apoptosis in cardiomyocytes. Bar = 100 μm. **b** The percentage of TUNEL-positive cells was determined and analyzed by ANOVA-LSD test. Significant difference was indicated: **, *P* < 0.01

We conducted the TUNEL assay to study the effect of RPostC treatment on the level of apoptosis in cardiomyocytes after reperfusion in an IRI rat model. As shown in Fig. 2 a-b, we observed an enhanced apoptotic signal in the IRI group compared with the control group (*P* < 0.01). However, a decreased apoptotic level in the IRI + RPostC group was detected when compared with that in the IRI group (Fig. 2 a-b, *P* < 0.01). Therefore, RPostC treatment in IRI rats is capable of attenuating the high myocardial apoptosis level induced by IRI.

### Gene expression profiling analysis

**Fig. 3:**
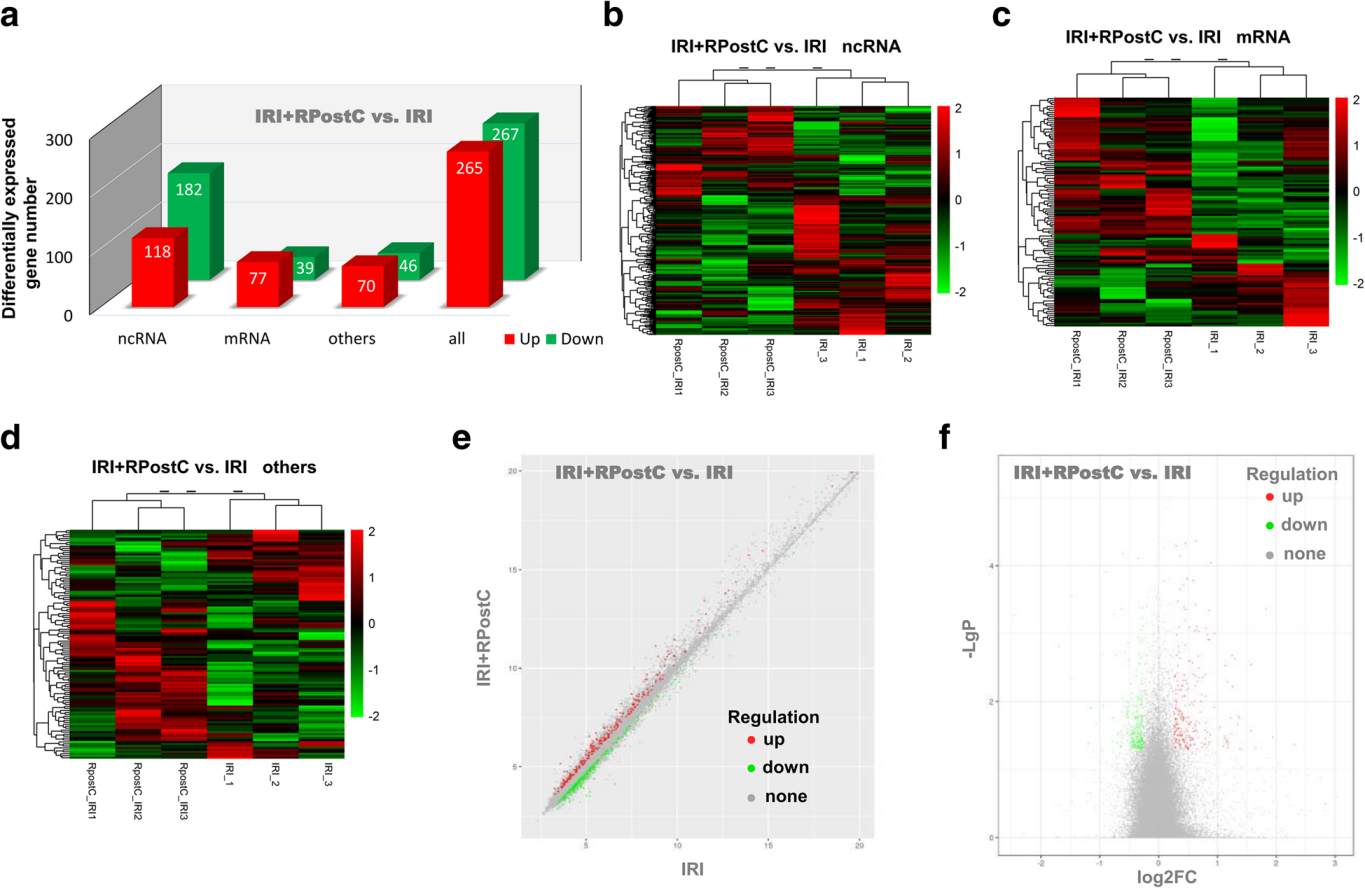
Genetic difference analysis data. a An Affymetrix Rat Gene 2.0 ST RNA microarray analysis was performed. Compared to expression in the IRI group, 265 upregulated genes and 267 downregulated genes were detected in the IRI + RPostC group. **b-d** The differentially expressed ncRNA, mRNA and others were subjected to hierarchical clustering and shown as a heatmap, respectively. The red signal indicates the upregulated genes, and the green signal indicates the downregulated genes. **e** The horizontal and vertical coordinates of scatter plot represent the log2 value of the expression levels of the two groups, respectively, showing the up-and-down distribution of the genes. **f** A volcano plot was also created based on the P- and fold-change (FC) values obtained by t-test analysis. The horizontal axis indicates the fold change of the probe, while the vertical axis represents the degree of difference in the probe (−log10 *P*-value, −LgP)

To investigate the molecular mechanism underlying the biological role of RPostC treatment, we conducted an Affymetrix Rat Gene 2.0 ST RNA microarray analysis using the above three rat models. Quality controls of microarray analysis are presented in Additional file 2: Figure S1. Compared to expression in the IRI group, 265 upregulated genes (with 118 ncRNA, 77 mRNA, 70 others) and 267 downregulated genes (with 182 ncRNA, 39 mRNA, 46 others) were detected in the IRI + RPostC group (Fig. 3a). To further comprehensively and intuitively present the difference between the two groups, the differentially expressed genes were subjected to hierarchical clustering and shown as a heatmap. The correlation between the samples was calculated based on the expression of the selected differential genes. Hierarchical cluster analysis data of noncoding RNAs (ncRNAs), mRNA and others are shown in Fig. 3 b-d. The red signal indicates the upregulated genes, and the green signal indicates the downregulated genes.

Additionally, Fig. 3e displays the scatter plot data. The horizontal and vertical coordinates represent the log2 value of the expression levels of the two groups, respectively, showing the up-and-down distribution of the genes. Furthermore, to show the significant differences between the two sets, a volcano plot (Fig. 3f) was created based on the *P*- and fold-change (FC) values obtained by t-test analysis. The horizontal axis indicates the fold change of the probe, while the vertical axis represents the degree of difference in the probe (−log10 *P*-value). We listed the top ten differentially upregulated ncRNA genes, which included MTA_TC0600002772.mm, MTA_TC1300002394.mm and MTA_TC1000001004.mm, in Table 1 and the downregulated ncRNAs, which included Rnu7, MTA_TC0500003037.mm and RGD7543256_1, in Table 2. More detailed information is shown in Additional file 3: Table S2. We also show a series of data from genetic difference analysis (Additional file 4: Fig. S2; Additional file 5: Table S3; Additional file 6: Table S4; Additional file 7: Table S5), hierarchical cluster analysis (Additional file 8: Figure S3), and scatter and volcano plots (Additional file 9: Figure S4) comparing IRI vs. control, IRI + RPostC vs. control and IRI + RPostC vs. IRI vs. control.

**Table 1:** The top ten differentially upregulated noncoding RNAs

Probe_set	FC	*P*.Value	Gene.Symbol	Description	Chromosome
TC1400001307.rn.1	2.9650765	0.0268688	MTA_TC0600002772.mm	Noncoding RNA, oocyte_clustered_small_RNA12319, complete sequence	chr14
TC0200002571.rn.1	2.5938385	0.0176662	MTA_TC1300002394.mm	Noncoding transcript identified by NONCODE: Sense No Exonic	chr2
TC2000000948.rn.1	2.5457357	0.0218726	MTA_TC1000001004.mm	Noncoding RNA, oocyte_clustered_small_RNA4900, complete sequence	chr20
TC0100003434.rn.1	2.2433064	0.0388949	MTA_TC1900000429.mm	Noncoding transcript identified by NONCODE: Antisense	chr1
TC0600001997.rn.1	2.1809429	0.0322078	MTA_TC0500000335.mm	Noncoding transcript identified by NONCODE: Sense No Exonic	chr6
TC1700001897.rn.1	1.9315169	0.0010048	MTA_TC1300000124.mm	microRNA 466i	chr17
TC0900000059.rn.1	1.9266048	0.0122216	RGD7738881_1	uncharacterized LOC102554115	chr9
TC1400000527.rn.1	1.8230086	0.0012326	MTA_TC0500002485.mm	Noncoding transcript identified by NONCODE: Linc	chr14
TC0300001341.rn.1	1.749724	0.0204293	RGD7622515_1	uncharacterized LOC102554631	chr3
TC1500001326.rn.1	1.7481686	0.0008483	MTA_TC1400000136.mm	Noncoding transcript identified by NONCODE: Sense No Exonic	chr15

*FC* fold change

**Table 2:** The top ten differentially downregulated noncoding RNAs

Probe_set	FC	*P*.Value	Gene.Symbol	Description	Chromosome
TC0900001686.rn.1	−5.481668	0.0005394	Rnu7	U7 small nuclear RNA	chr9
TC1200000831.rn.1	−2.788872	0.0045801	MTA_TC0500003037.mm	RNA for germline small RNA gsRNA59, complete sequence	chr12
TC0400002518.rn.1	−2.773747	0.0002789	RGD7543256_1	uncharacterized LOC102551678	chr4
TC0300003185.rn.1	−2.291115	0.0410185	MTA_TC0200003621.mm	predicted gene, 22,403	chr3
TC0400001897.rn.1	−2.076163	0.0211123	MTA_TC0600001556.mm	nuclear encoded rRNA 5S 166	chr4
TC0200004714.rn.1	−1.948049	0.0019727	RGD7555511_1	uncharacterized LOC102553185	chr2
TC0300004193.rn.1	−1.917423	0.0114548	MTA_TC0200004648.mm	microRNA 3098 (Mir3098), microRNA	chr3
TC0X00001589.rn.1	−1.850113	0.0001236	MTA_TC0X00001571.mm	predicted gene, 22,359	chrX
TC0100006120.rn.1	−1.723291	0.0389854	MTA_TC0700003802.mm	microRNA 3965, microRNA 3965 (Mir3965), microRNA	chr1
TC1300000971.rn.1	−1.722596	0.0173776	Mir664–2	microRNA mir-664-2	chr13

*FC* fold change

### GO enrichment and functional pathway analysis

**Fig. 4:**
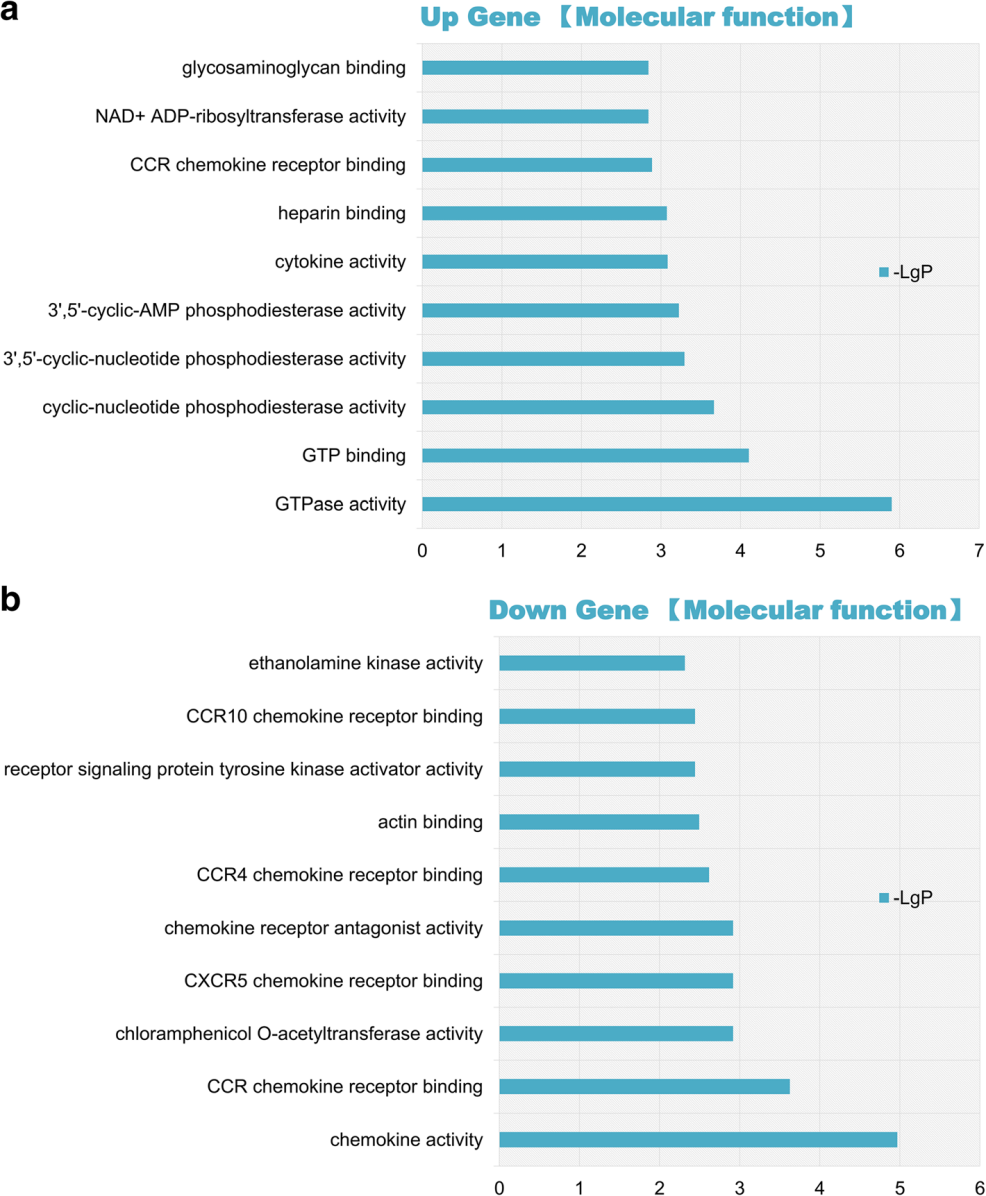
GO Enrichment analysis of molecular function. To determine the significant, accurate, targeted gene functions of the target genes, GO enrichment analysis was performed. A group of upregulated genes (**a**) and downregulated genes (**b**) associated with molecular functions were shown. The vertical axis indicates the pathway names, while the horizontal axis represents the degree of difference in the probe (−log10 *P*-value, −LgP)

Next, we performed GO enrichment analysis to determine the significant, accurate, targeted gene functions of the target genes. As shown in Fig. 4a, Additional file 10: Figure S5a, and Additional file 11: Figure S6a, we found a group of upregulated genes associated with molecular functions such as GTPase activity, GTP binding, cyclic nucleotide phosphodiesterase activity, cytokine activity, the cellular response to interferon-beta, and symbiont-containing vacuole member, which is likely to be involved in the myocardial protection by RPostC during ischemia/reperfusion injury. Moreover, downregulated genes associated with molecular functions, such as chemokine activity, CCR chemokine receptor binding, the positive regulation of cell-cell adhesion mediated by integrin, and the formation of stress fibers, were also detected (Fig. 4b, Additional file 10: Figure S5b, and Additional file 11: Figure S6b).

**Fig. 5:**
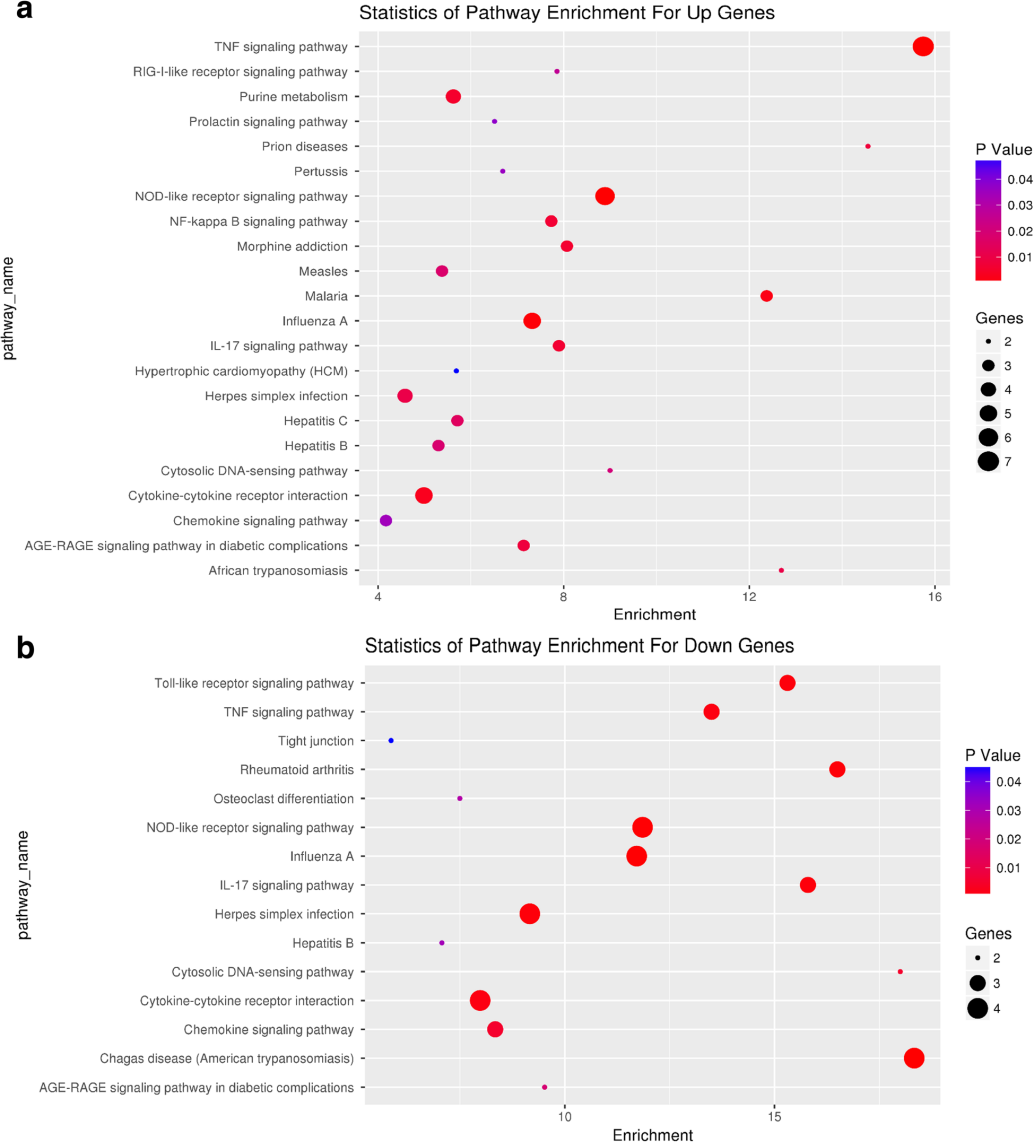
Statistics of pathway enrichment. When comparing the IRI group with the IRI + RPostC group, the relative KEGG pathways were identified, using the above upregulated genes (**a**) and downregulated genes (**b**). The vertical axis indicates the GO terms, while the horizontal axis provides the degree of enrichment. Size of the dot represents the number of enriched genes, and the color of the dot represents the *P* value

Based on the bioinformatics resource KEGG, we also carried out pathway enrichment analysis. As shown in Fig. 5, when comparing the IRI group with the IRI + RPostC group, the TNF signaling pathway and Toll-like receptor signaling pathway were identified, indicating that these pathways may be involved in the cardioprotective role of RPostC.

### Network analysis

**Fig. 6:**
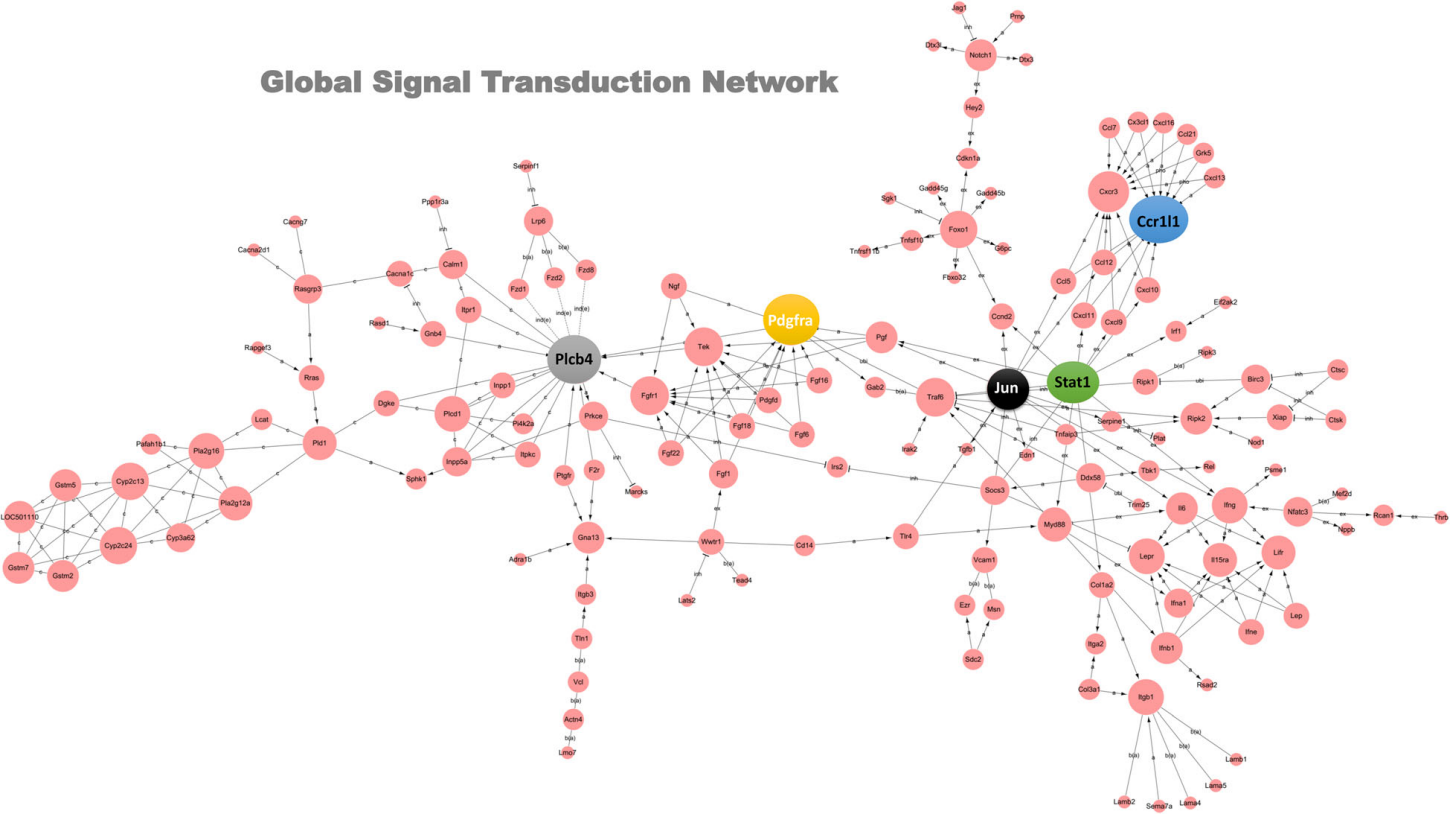
Global signal transduction network data. Based on the differentially expressed gene data, we performed a global signal transduction network analysis to identify a total of 202 core genes, including *Plcb4*, *Pdgfra*, *Ccr1 l1*, *Stat1* and *Jun*.

To further identify the potential core genes, which are essential in signaling networks and RNA-RNA interactions, we performed a global signal transduction network analysis and co-expression network analysis. The data from the global signal transduction network analysis (Fig. 6 and Additional file 12: Table S6) identified a total of 202 core genes, including *Plcb4*, *Pdgfra*, *Ccr1 l1*, *Stat1* and *Jun*. In addition, the data from the co-expression network analysis (Additional file 13: Figure S7 and Additional file 14: Table S7) identified 436 core genes, including *Hba2*, *LOC501110*, *Spag9*, *Stfa3* and *Cflar*. Moreover, we performed a Venn diagram analysis to obtain a total of 64 common genes (Additional file 15: Figure S8a). We further limited the network degree value to five, and three genes, including *LOC501110*, *Lifr* and *Gstm5*, were identified (Additional file 15: Figure S8b).

### Quantitative real-time PCR analysis

**Fig. 7:**
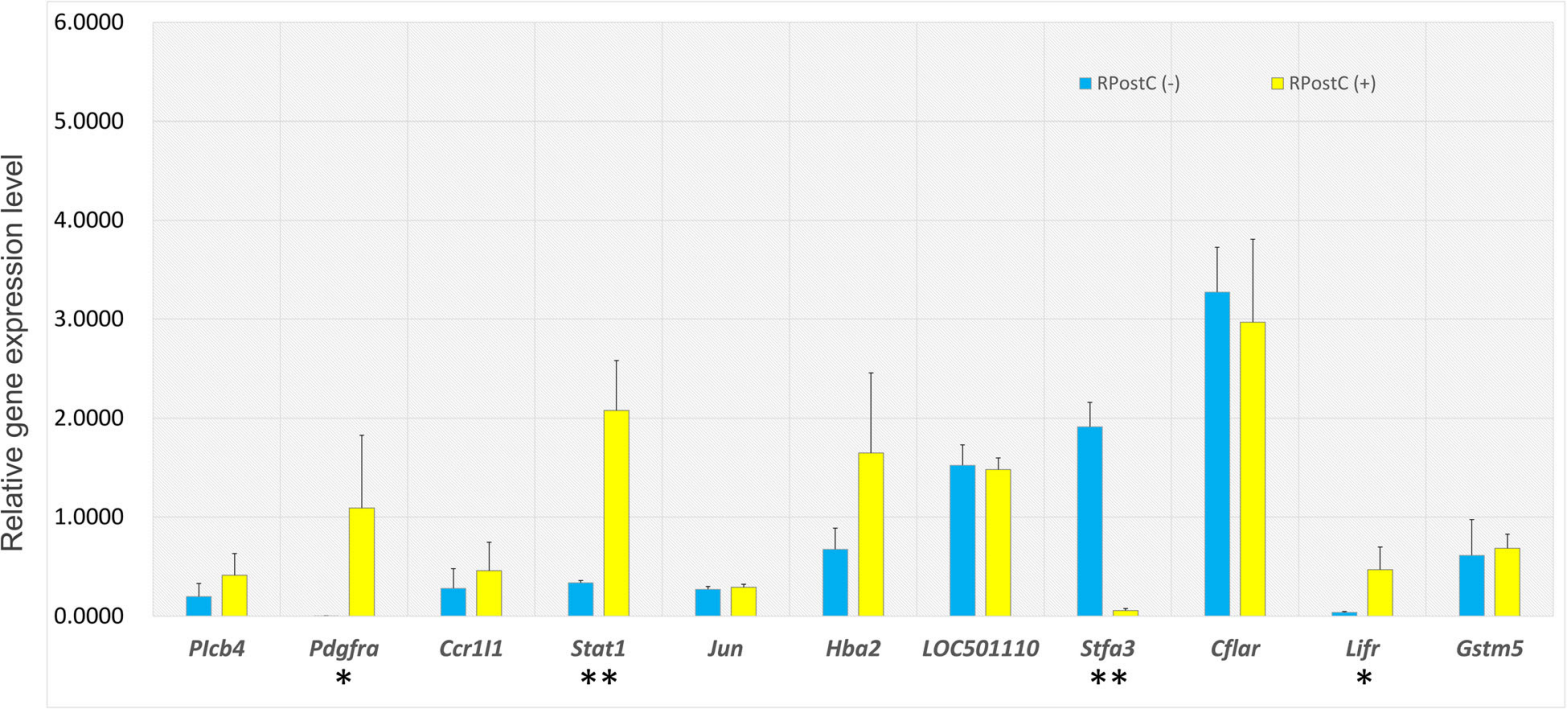
qPCR analysis data targeting several genes. To further verify the differentially expressed mRNAs, a qPCR assay was performed. The 2^-ΔΔCT^ method was applied to calculate the relative transcript levels. The relative expression of *Gapdh* was utilized to normalize the expression of tested genes, including *Plcb4*, *Pdgfra*, *Ccr1l1*, *Stat1*, *Jun*, *Hba2*, *LOC501110*, *Stfa3*, *Cflar*, *Lifr*, and *Gstm5*. Independent sample Student’s t-test was also performed for the statistical analysis. Significant difference was indicated: *, *P* < 0.05; **, *P* < 0.01

We also performed quantitative real-time PCR analysis to further verify the differentially expressed mRNAs. As shown in Fig. 7, the upregulation of genes including *Pdgfra*, *Stat1* and *Lifr* was detected, while the downregulation of the *Stfa3* gene was observed. Further experiments targeting these genes are required.

## Discussion

The protective role of ischemic preconditioning and postconditioning, to varying degrees, has previously been identified in several studies using animal models. For example, it has been shown that ischemic preconditioning treatment in myocardial IRI rats exhibits a more powerful protective effect than limb remote ischemic postconditioning treatment during myocardial ischemia-reperfusion injury [22]. Compared to classic postconditioning, remote postconditioning treatment has a greater potential role in reducing the infarct size in a New Zealand white male rabbit model [23]. In the present study, we utilized an in vivo rat model of IRI to evaluate the role of RPostC treatment after myocardial ischemia-reperfusion injury. We found that RPostC treatment resulted in a reduction in the IRI-induced myocardial infarction area. The TUNEL assay data further indicated the involvement of an antiapoptotic effect in the protective mechanism of RPostC treatment after myocardial injury. These data are consistent with relevant studies in other animal models. For instance, myocardial infarct size was also significantly decreased in the RPostC group compared with the control group after acute myocardial infarction in pigs [24].

The molecular mechanism of the protective effect of RPostC treatment remains largely unclear. Multiple processes, such as the inflammatory response, oxidative stress, and leukocyte infiltration, and gasotransmitters (NO, H_2_S and CO) may be potentially implicated in the role of RPostC in myocardial ischemia-reperfusion injury [9, 13, 25–29]. For example, the inhibition of receptors for advanced glycation end products (RAGE) and high-mobility group box1 (HMGB1) expression and activation of the PI3K/Akt signaling pathway have been linked to the extenuated ischemic reperfusion injury in a mouse model [29]. Our current study revealed that chemokine activity, CCR chemokine receptor binding and cytokine activity may be associated with the myocardial protective role of RPostC treatment after ischemia/reperfusion injury in a rat model, which supports the idea that the inflammatory response is indeed an essential factor for reducing ischemia-reperfusion-induced cardiac damage. Moreover, our findings offer a potential functional link between GTPase activity, GTP binding issues and cardioprotection by RPostC treatment.

The Toll-like receptor signaling pathway is related to the myocardial immune response and ischemia/reperfusion [30–33]. It has been reported that the postconditioning of sevoflurane confers a neuroprotective role in a rat model of transient global cerebral ischemia, and the Toll-like receptor-4 (TLR4)/nuclear factor kappa B (NF-κB) pathway and subsequent anti-inflammation activity may be implicated in this process [34]. The TLR4/NF-κB signaling pathway has also been reported to be associated with vaspin-mediated cardioprotective effects on myocardial ischemia/reperfusion injury [35]. Herein, we further utilized an Affymetrix Rat Gene 2.0 ST chip to perform gene expression profiling, GO enrichment and functional pathway analysis. We also observed the potential role of the Toll-like receptor signaling pathway in the RPostC-mediated cardioprotective process.

Additionally, it is worth mentioning that a we are the first to identify a group of differentially expressed ncRNA genes. Our global signal transduction network analysis and co-expression network analysis also identified several core genes, such as *Pdgfra*, *Stat1, Lifr* and *Stfa3.* It is meaningful to study the expression of RPostC-associated ncRNAs and core genes and to investigate their functional relationship with the serum or myocardial level of inflammatory-related biomarkers, which may contribute to the optimization of the details of the RPostC protocol and an increase in survival duration. Considering the role of coronary circulation in the cardioprotection process [36], more relative molecular investigations are needed to further study the functional role of the expression of the targeting genes in the cardiomyocytes and other protected cardiac cells.

Ischemic postconditioning treatment exhibits a protective role on the heart as well as some other ischemia-sensitive organs via a complex mechanism. For instance, RPostC treatment has an influence on cerebral ischemia-reperfusion injury [37–39]. The T-LAK-cell-originated protein kinase (TOPK)/phosphatase and tensin homolog deleted on chromosome ten (PTEN)/Akt signaling pathway is associated with the protective effects of RPostC treatment after renal ischemia/reperfusion injury [40]. RPostC treatment exhibits a protective effect on limb ischemia-reperfusion-induced gastric mucosal injury, in which it is implicated in anti-inflammatory and antioxidant activity [41]. Ischemic postconditioning treatment also reportedly has a protective effect on renal ischemia and reperfusion injury in rats via the modulation of the anti-inflammatory response [42] and on the ischemia-reperfusion injury of rat liver graft [43]. The hypoxia inducible factor 1 alpha (HIF-1α)/microRNA-21 (miR-21) axis may contribute to the protective role of the ischemic postconditioning approach [44]. Although remote ischemic postconditioning treatment is a feasible operation strategy, it is difficult to control the degree and timing of ischemic postconditioning intervention. The myocardial protection process triggered by RPostC treatment is closely associated with the length of ischemic and intervention time and the physiological features of different ischemic tissues and organs.

The combination of natural pharmaceuticals, such as troxerutin, and RPostC treatment has also been considered [27]. Support for pharmacological and/or ischemic postconditioning requires additional clinical trial data [45]. Postconditioning of endomorphin-1 in a rat model also reportedly decreases myocardial cell apoptosis and tissue injury, possibly through the regulation of inflammatory or oxidative stress [46]. All of these methods will be useful to reduce IRI-mediated postoperative complications. Given the complicated mechanism of myocardial damage after ischemia and reperfusion [47, 48], additional molecular evidence regarding the role of RPostC in IRI-induced myocardial necrosis or pyroptosis is needed. In addition, considering the critical issues for the translation from experimental cardioprotection studies to clinical patient benefit [49], more results of the clinical trials or molecular tests from the patients are required to support our gene expression profiling analysis data from the rat models.

## Conclusions

To ensure optimal myocardial protection and minimize other influencing factors, it is meaningful to investigate the specific molecular mechanisms underlying remote ischemic postconditioning, optimize specific clinical implementation methods, discover relevant biomarkers, develop related drugs, and target the drugs to their corresponding signal transduction pathways. The potential protective effect of RPostC and its association with various ncRNAs, GTPase activity, cytokine activity, the TNF and Toll-like receptor signaling pathways, and core genes such as *Pdgfra*, *Stat1, Lifr* and *Stfa3* merit further molecular analysis.

## Additional files

Additional file 1: **Table S1.** The primer sequences used in the qPCR assay. (XLSX 31 kb)

Additional file 2: **Figure S1.** Quality controls of the microarray analysis. **a** Chip-box data; **b** chip-histogram data; **c** hybrid quality control; **d** negative-positive quality control. (TIF 7165 kb)

Additional file 3: **Table S2**. The differently expressed genes identified by IRI + RPostC vs. IRI vs. control comparison. (XLSX 203 kb)

Additional file 4: **Figure S2.** An Affymetrix Rat Gene 2.0 ST RNA microarray analysis was performed for the genetic difference analysis data. A number of upregulated genes and downregulated genes were detected in the comparisons of IRI vs. control (**a**); IRI + RPostC vs. control (**b**); IRI + RPostC vs. IRI vs. control (**c (TIF 8798 kb)**

Additional file 5: **Table S3**. The differently expressed genes identified by IRI vs. control comparison. (XLSX 2576 kb)

Additional file 6: **Table S4.** The differently expressed genes identified by IRI + RPostC vs. control comparison. (XLSX 3221 kb)

Additional file 7: **Table S5.** The differently expressed genes identified by IRI + RPostC vs. control comparison. (XLSX 3603 kb)

Additional file 8: **Figure S3.** The differentially expressed ncRNA, mRNA and others in the comparisons of IRI vs. control (**a-c**), IRI + RPostC vs. control (**d-e**), IRI + RPostC vs. IRI vs. control (**f-h**) were subjected to hierarchical clustering and shown as a heatmap, respectively. The red signal indicates the upregulated genes, and the green signal indicates the downregulated genes. (TIF 17130 kb)

Additional file 9: **Figure S4.** The horizontal and vertical coordinates of scatter plots in the comparisons of IRI vs. control (**a**), IRI + RPostC vs. control (**b**) represent the log2 value of the expression levels of the two groups, respectively, showing the up-and-down distribution of the genes. The volcano plots in the comparisons of IRI vs. control (**c**), IRI + RPostC vs. control (**d**) was also created based on the *P*- and fold-change (FC) values obtained by t-test analysis. The horizontal axis indicates the fold change of the probe, while the vertical axis represents the degree of difference in the probe (−log10 *P*-value, −LgP). (TIF 33038 kb)

Additional file 10: **Figure S5.** GO Enrichment analysis of biological processes. A group of upregulated genes (**a**) and downregulated genes (**b**) associated with biological processes were shown. The vertical axis indicates the pathway names, while the horizontal axis represents the degree of difference in the probe (−log10 *P*-value, −LgP). (TIF 48577 kb)

Additional file 11: **Figure S6.** GO Enrichment analysis of cellular components. A group of upregulated genes (**a**) and downregulated genes (**b**) associated with cellular components were shown. The vertical axis indicates the pathway names, while the horizontal axis represents the degree of difference in the probe (−log10 *P*-value, −LgP). (TIF 52073 kb)

Additional file 12: **Table S6.** Gene information from the global signal transduction network analysis. (XLSX 37 kb)

Additional file 13: **Figure S7.** Based on the normalized signal intensity of RNA expression, co-expression network analysis was performed to detect potential correlations among mRNAs and identify the core genes by the degree of differences. (TIF 31248 kb)

Additional file 14: **Table S7.** Gene information from the co-expression network analysis. (XLSX 67 kb)

Additional file 15: **Figure S8.** The Venn diagram analysis data. **a** All core genes identified by global signal transduction network analysis and co-expression network analysis. **b** All core genes identified by the two network analyses with a degree number > =5. (TIF 12766 kb)

## Abbreviations

IRI: Ischemia reperfusion injury RPostC Remote ischemic postconditioning ncRNAs non-coding RNAs Rnu7 U7 small nuclear RNA PostC Ischemic postconditioning LAD Left anterior descending coronary artery LFA Left femoral artery TTC 2,3,5-triphenylte-trazolium chloride PBS Phosphate buffer saline TUNEL Terminal dexynucleotidyl transferase (TdT)-mediated dUTP nick end labeling CLV Cardiac left ventricle GO Gene Ontology KEGG Kyoto Encyclopedia of Genes and Genomes NCBI National center for biotechnology information qPCR quantitative real-time polymerase chain reaction assay Gapdh Glyceraldehyde-3-Phosphate Dehydrogenase Plcb4 Phospholipase C, beta 4 Pdgfra Platelet derived growth factor receptor alpha Ccr1l Chemokine (C-C motif) receptor 1-like 1 Stat1 Signal transducer and activator of transcription 1 Jun Jun proto-oncogene,AP-1 transcription factor subunit Hba2 Hemoglobin Subunit Alpha 2 LOC501110 Similar to Glutathione S-transferase A1 (GTH1) (HA subunit 1) (GST-epsilon) (GSTA1–1) (GST class-alpha) Stfa3 Stefin A3 Cflar CASP8 and FADD-like apoptosis regulator Lifr Leukemia inhibitory factor receptor alpha Gstm5 Glutathione S-transferase, mu 5 ANOVA One-way analysis of variance LSD Least Significant Difference FDR False positive rate FC Fold change RAGE Inhibited receptor for advanced glycation end products HMGB1 High-mobility group box1 TLR4 Toll-Like Receptor-4 NF-κB Nuclear Factor Kappa B TOPK T-LAK-cell-originated protein kinase PTEN Phosphatase and tensin homolog deleted on chromosome ten HIF-1α Hypoxia inducible factor 1 alpha miR-21 microRNA-21
