# Supplementary figures and images for: Gene expression profiling analysis to investigate the role of remote ischemic postconditioning in ischemia-reperfusion injury in rats
Source: BMC Genomics. 2019 May 9;20:361. doi: 10.1186/s12864-019-5743-9 (PMC6509872; doi:10.1186/s12864-019-5743-9)

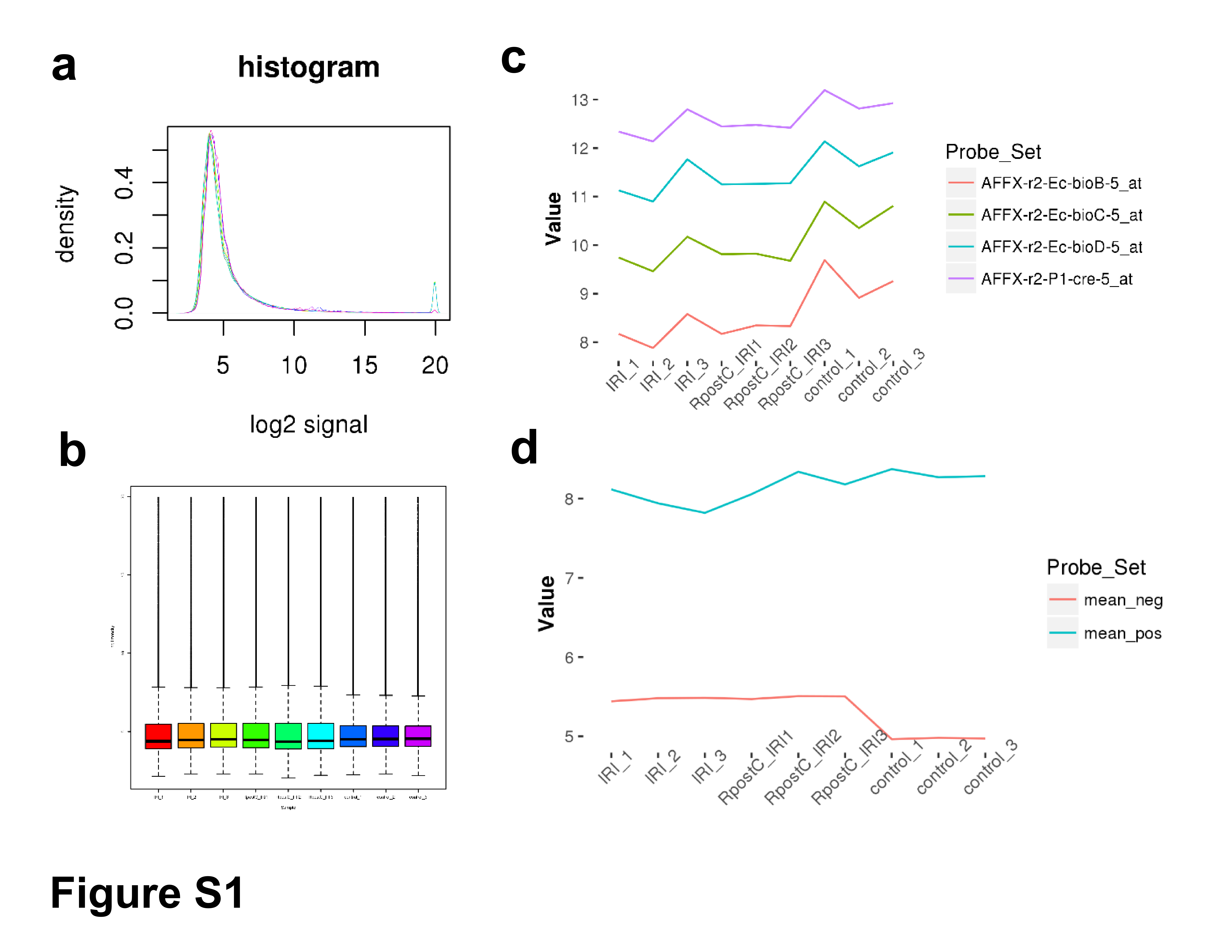

Supplement: Supplementary file 2 — Figure S1. Quality controls of the microarray analysis. a Chip-box data; b chip-histogram data; c hybrid quality control; d negative-positive quality control. (TIF 7165 kb) [file 12864_2019_5743_MOESM2_ESM.tif]

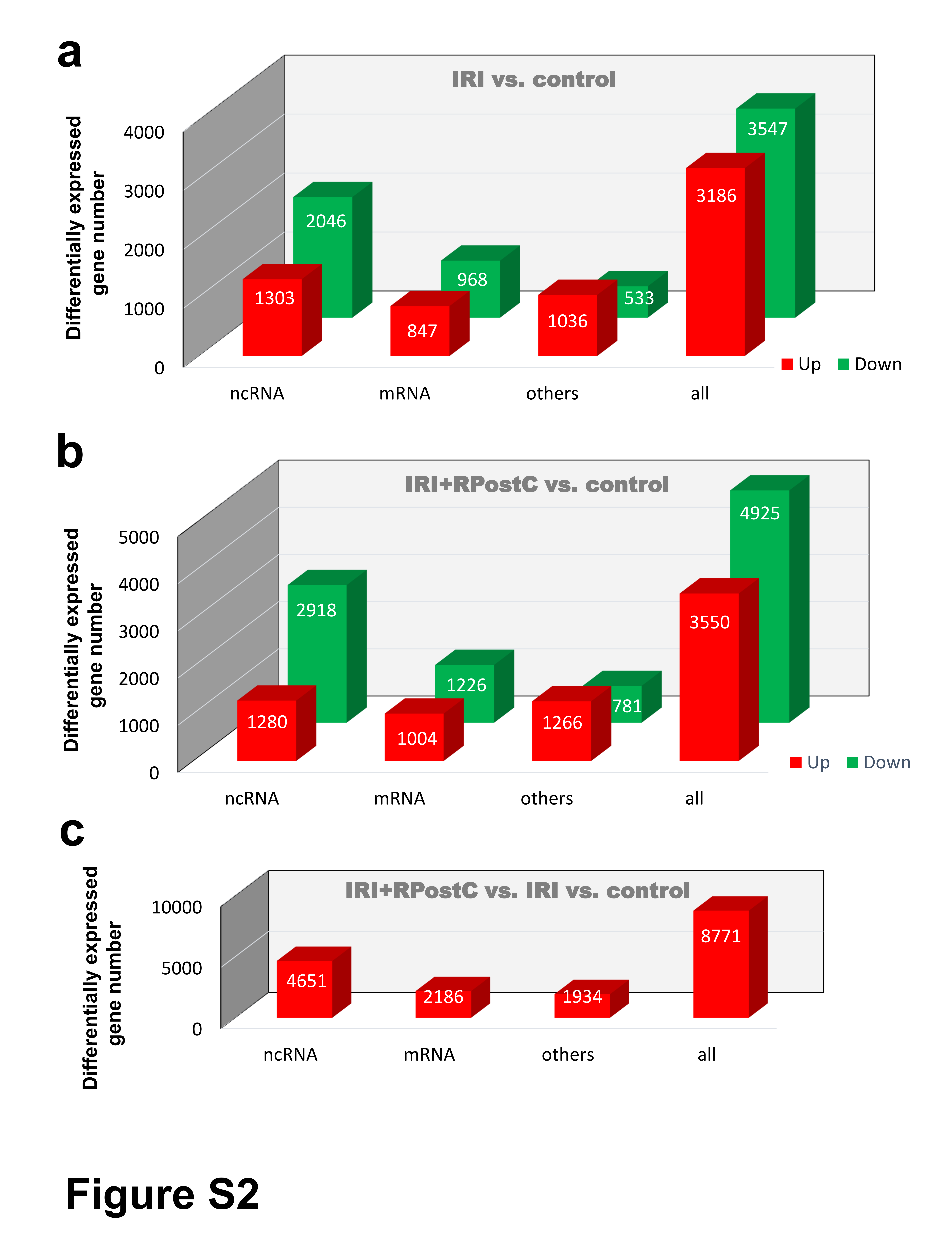

Supplement: Supplementary file 4 — Figure S2. An Affymetrix Rat Gene 2.0 ST RNA microarray analysis was performed for the genetic difference analysis data. A number of upregulated genes and downregulated genes were detected in the comparisons of IRI vs. control (a); IRI + RPostC vs. control (b); IRI + RPostC vs. IRI vs. control (c (TIF 8798 kb) [file 12864_2019_5743_MOESM4_ESM.tif]

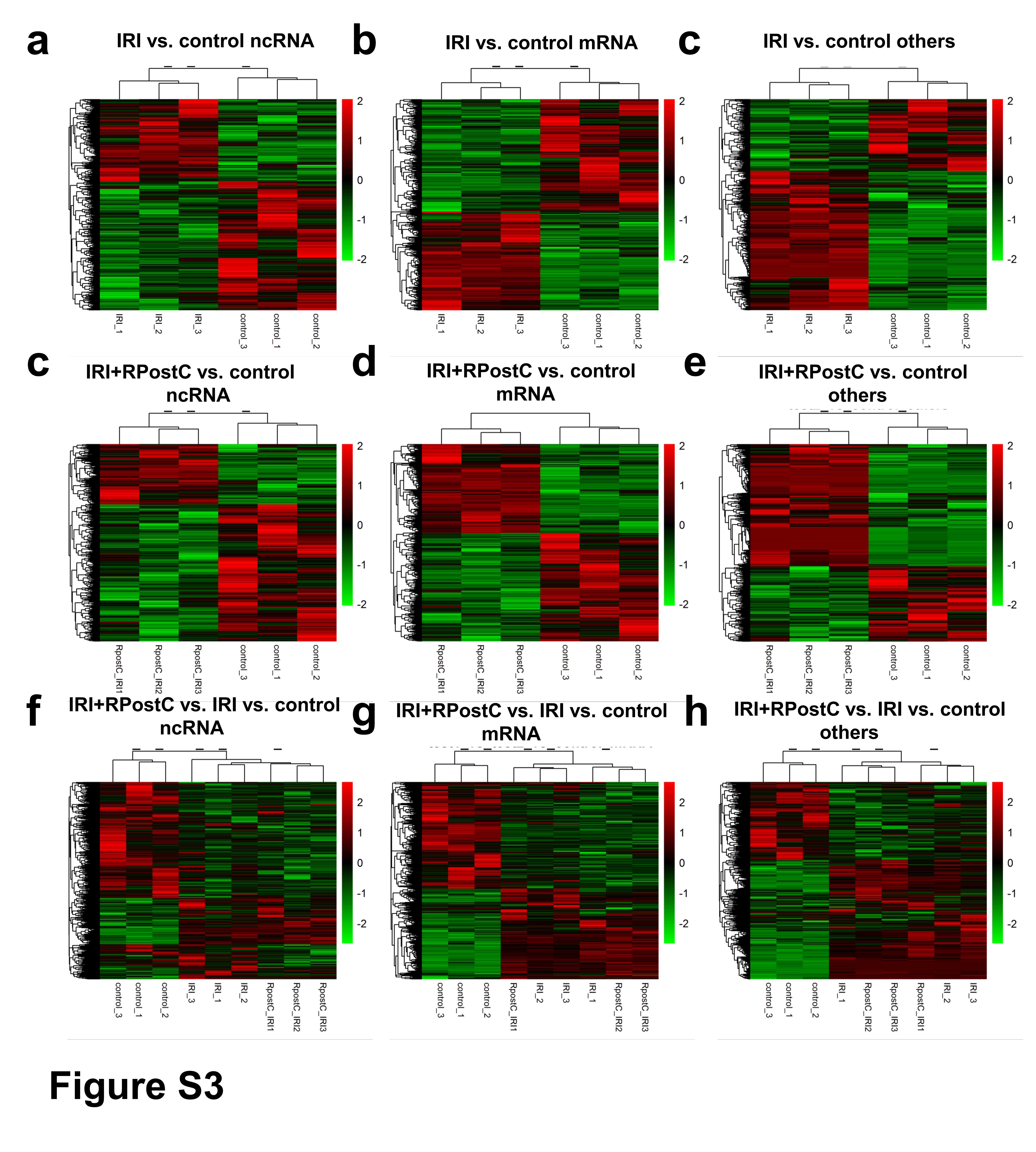

Supplement: Supplementary file 8 — Figure S3. The differentially expressed ncRNA, mRNA and others in the comparisons of IRI vs. control (a-c), IRI + RPostC vs. control (d-e), IRI + RPostC vs. IRI vs. control (f-h) were subjected to hierarchical clustering and shown as a heatmap, respectively. The red signal indicates the upregulated genes, and the green signal indicates the downregulated genes. (TIF 17130 kb) [file 12864_2019_5743_MOESM8_ESM.tif]

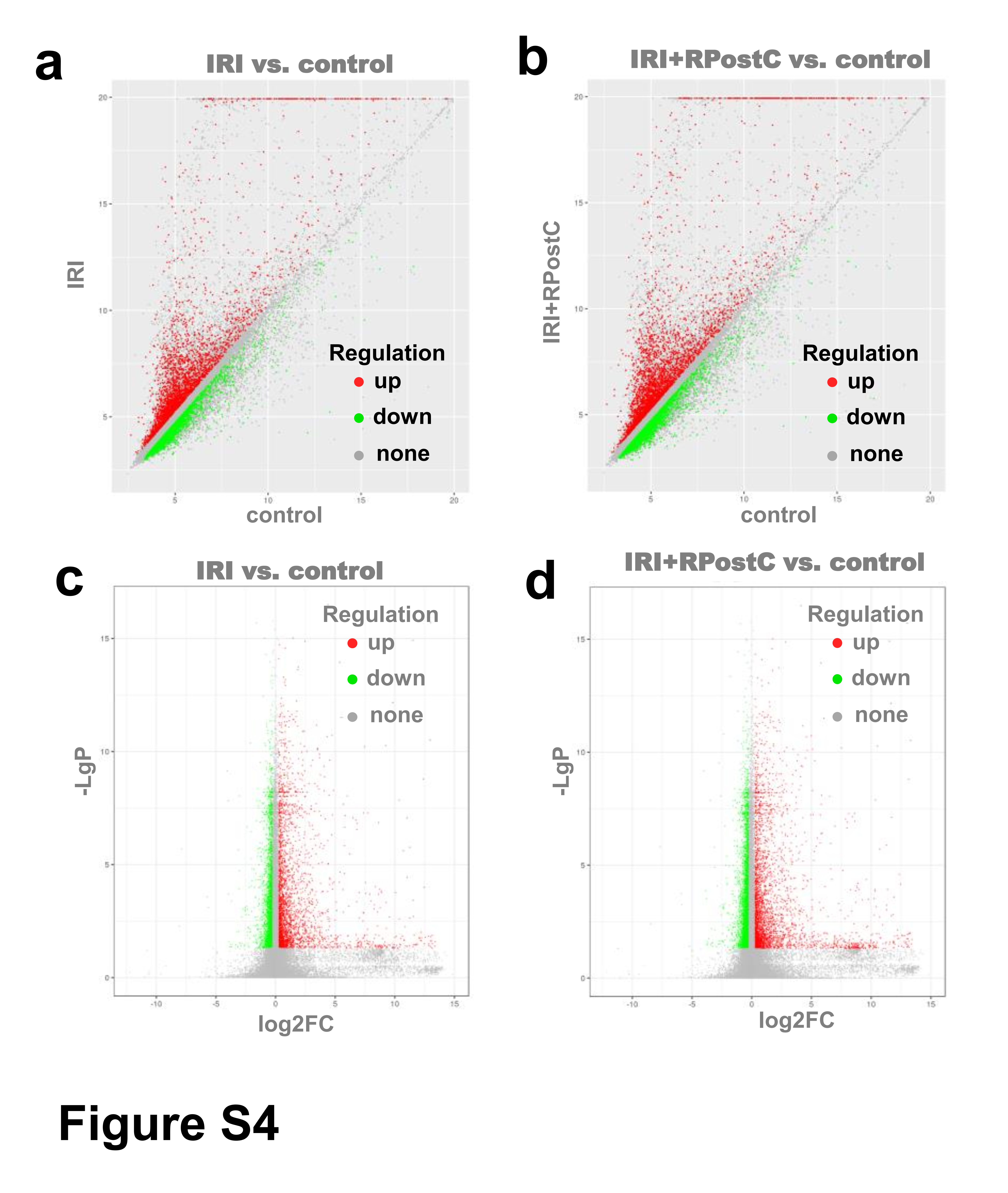

Supplement: Supplementary file 9 — Figure S4. The horizontal and vertical coordinates of scatter plots in the comparisons of IRI vs. control (a), IRI + RPostC vs. control (b) represent the log2 value of the expression levels of the two groups, respectively, showing the up-and-down distribution of the genes. The volcano plots in the comparisons of IRI vs. control (c), IRI + RPostC vs. control (d) was also created based on the P- and fold-change (FC) values obtained by t-test analysis. The horizontal axis indicates the fold change of the probe, while the vertical axis represents the degree of difference in the probe (−log10 P-value, −LgP). (TIF 33038 kb) [file 12864_2019_5743_MOESM9_ESM.tif]

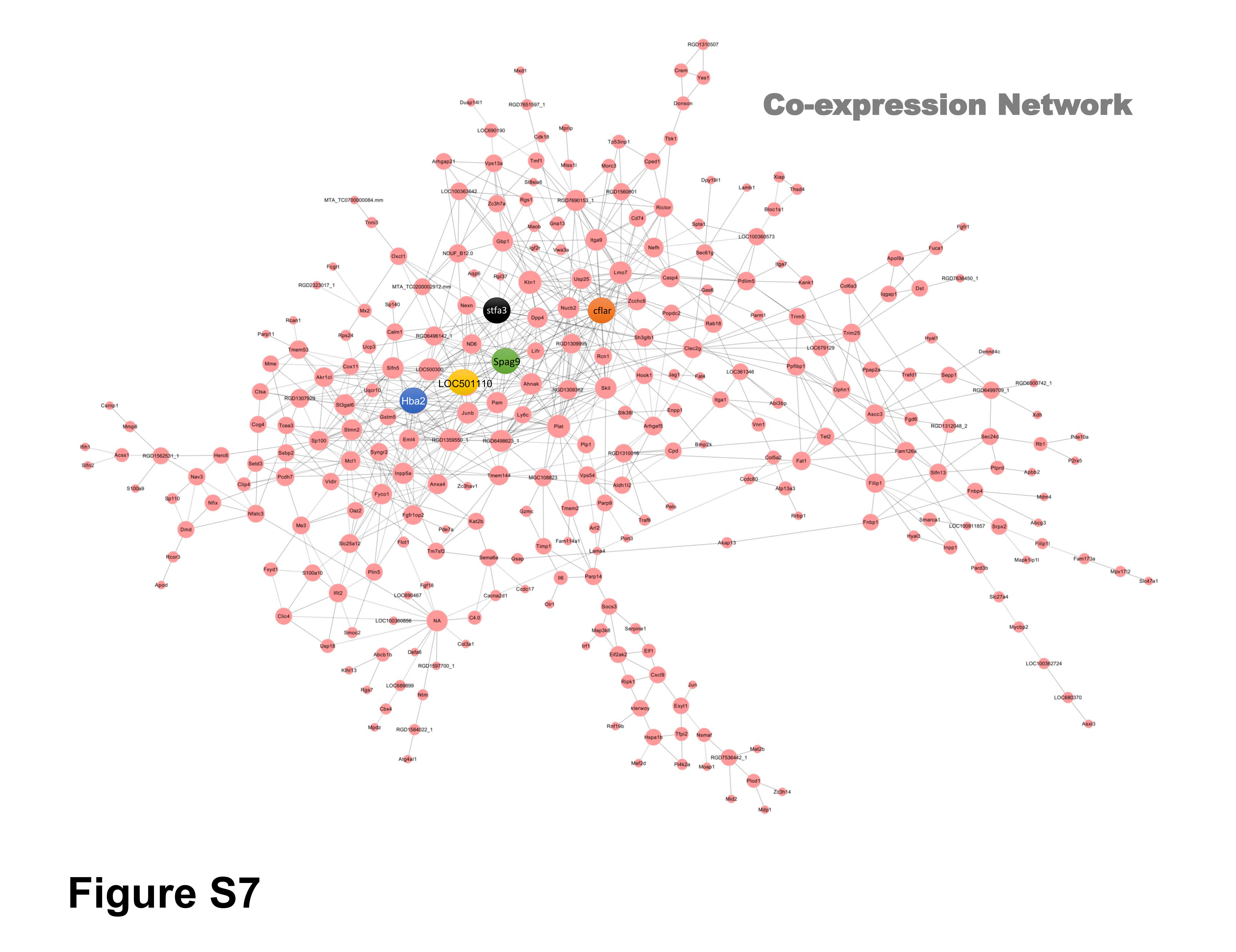

Supplement: Supplementary file 13 — Figure S7. Based on the normalized signal intensity of RNA expression, co-expression network analysis was performed to detect potential correlations among mRNAs and identify the core genes by the degree of differences. (TIF 31248 kb) [file 12864_2019_5743_MOESM13_ESM.tif]
